# Lipoteichoic acid of *Enterococcus faecalis* inhibits osteoclastogenesis via transcription factor RBP-J

**DOI:** 10.1177/1753425918812646

**Published:** 2018-11-21

**Authors:** Shuai Wang, Boon Chin Heng, Shuqi Qiu, Jing Deng, Gary Shun Pan Cheung, Lijian Jin, Baohong Zhao, Chengfei Zhang

**Affiliations:** 1 Department of Stomatology, The Affiliated Hospital of Qingdao University, School of Stomatology of Qingdao University, Qingdao, China; 2 Endodontology, Faculty of Dentistry, The University of Hong Kong, Hong Kong SAR, China; 3 HKU Shenzhen Institute of Research and Innovation, Shenzhen, China; 4 Shenzhen Key Laboratory of ENT, Institute of ENT & Longgang ENT hospital, Shenzhen, China; 5 Periodontology, Faculty of Dentistry, The University of Hong Kong, Hong Kong SAR, China; 6 Arthritis and Tissue Degeneration Program, and David Z. Rosensweig Genomics Research Center, Hospital for Special Surgery, New York, USA; 7 Department of Medicine, Weill Cornell Medical College, New York, USA

**Keywords:** *Enterococcus faecalis*, lipoteichoic acid, bone marrow-derived macrophages, osteoclastogenesis, RBP-J

## Abstract

Lipoteichoic acid (LTA) of *Enterococcus faecalis* is a potent stimulator of inflammatory responses, but the effects of *E. faecalis* LTA on osteoclastogenesis remains far from well understood. This study showed that *E. faecalis* LTA significantly inhibited osteoclastogenesis of wild type murine bone marrow-derived macrophages (BMMs) in the presence of a high dose of RANKL, while the inhibition of osteoclastogenesis by *E. faecalis* LTA was significantly removed in BMMs with deficient expression of the transcription factor RBP-J. In addition, a few small osteoclasts were generated in BMMs with only *E. faecalis* LTA stimulation, presumably due to the production of TNF-α and IL-6. Furthermore, both p38 and ERK1/2 MAPK signaling pathways were activated after 24 h of *E. faecalis* LTA treatment, but these signaling pathways were not activated after 6 d of treatment with RANKL in mature osteoclasts. In conclusion, *E. faecalis* LTA, which induces inflammatory response, could inhibit RANKL-induced osteoclastogenesis via RBP-J in BMMs.

## Introduction

Lipoteichoic acid (LTA) is an amphiphile which is located at the interface of the cytoplasmic membrane and cell wall of pathogenic and non-pathogenic Gram-positive bacteria and is released during growth.^1^ LTA, as a major virulence factor, plays an important role in stimulation of inflammatory responses.^2^ The predominant virulent attributes of *Enterococcus faecalis* consist of lytic enzymes, cytolysin, aggregation substance, peptidoglycan and LTA which can promote colonization, invasion of host tissues and evasion of host defense mechanisms.^3^ *E. faecalis* LTA can induce inflammatory responses by stimulating macrophages to release cytokines and mediators,^4^ and contributes to biofilm formation that enables bacteria survival in adverse environments.^5^

Bone homeostasis is a dynamic balance between bone resorption and bone formation.^6^ Disruption of the balance between osteoblasts and osteoclasts will result in osteopenia/osteoporosis or other metabolic bone diseases.^7^ Bone resorptive osteoclasts are multinucleated cells derived from monocyte/macrophage precursors.^8^ The receptor activator of RANKL and M-CSF are two essential cytokines for osteoclast differentiation.^9^ When pro-inflammatory cytokines stimulate osteoclast precursors, the transcription factors NF-κB, c-Fos and NFATc1, which play essential roles during osteoclast differentiation, will be further activated.^10–12^ NFATc1 modulates osteoclast-specific genes including cathepsin K, TRAP and matrix metalloproteinase-9 (MMP-9).^13–15^

The transcription factor recombinant recognition sequence binding protein at the J_κ_ site (RBP-J) is expressed in most cells and is a nuclear DNA-binding protein that can repress or activate transcription when acting in conjunction with different proteins.^16^ RBP-J is involved in cell proliferation, differentiation and cell fate determination.^16^ RBP-J plays an important role in pro-inflammatory M1 macrophage polarization.^17^ Both NK-κB and Notch signaling pathways are associated with osteoclastogenesis.^18^ The induction of NFATc1 is dependent on NF-κB and c-Fos pathways resulting in osteoclast differentiation. RBP-J negative regulates the expression and function of NFATc1 via inhibition of NF-κB and c-Fos and further suppresses osteoclast differentiation and bone resorption.^18^^,^^19^ On the other hand, the Notch signaling pathway participates in bone remodeling, and the activation of the Notch intracellular domain 1 (NICD1) significantly activates RBP-J activity.^19^ When Notch signaling is attenuated, osteoclastogenesis and bone resorption will be aggravated.^20^ It was reported that RBP-J negatively regulates osteoclast differentiation and bone resorption, particularly in TNF-α-induced osteoclastogenesis and inflammatory bone resorption.^18^^,^^19^ It has also been found that TNF or LPS-mediated osteoclast differentiation and inflammatory bone resorption are drastically suppressed by RBP-J and IFN regulatory factor-8.^18^^,^^21^ These studies show that RBP-J has a strong inhibitory effect on osteoclast differentiation and inflammatory bone resorption.

To date, the effects of *E. faecalis* LTA on osteoclast differentiation within the inflammatory environment of persistent apical periodontitis caused by *E. faecalis* is still unclear. Hence, in this study, we explored the modulatory effects and mechanisms of *E. faecalis* LTA on the differentiation of inflammatory osteoclasts and the relevant underlying mechanisms involved.

## Materials and methods

### Bacterial culture and LTA preparation

*E. faecalis* P25RC and P52Sa were isolated, respectively, from patients’ root canal and saliva at the Hospital of Stomatology of Peking University by Dr. Xiaofei Zhu.^22^ *E. faecalis* ATCC 29212 was purchased from the American Type Culture Collection (ATCC, Manassas, VA, USA). *E. faecalis* were cultured anaerobically (N_2_, 90%, CO_2_, 5% and H_2_, 5%) overnight at 37°C in brain heart infusion broth (OXOID, Basingstoke, Hampshire, England). The three highly purified *E. faecalis* LTAs were extracted using the butanol method followed by hydrophobic interaction chromatography purification. Contaminations were excluded as described in our previous paper.^23^

### Culture of osteoclast precursors

Bone marrow-derived macrophages (BMMs) from wild type (WT) and *Rbpj* conditional knockout mice (*Rbpj^ΔM/ΔM^*) were used as osteoclast precursors as previously described.^18^ Cells were cultured to 80% confluence, and then treated with the three *E. faecalis* LTAs (50 μg/ml), RANKL (80 ng/ml), *E. faecalis* LTAs (50 μg/ml) plus RANKL (80 ng/ml) for 6 d, or pre-treated with RANKL (20 ng/ml) for 3 d prior to treatment with *E. faecalis* LTAs (50 ng/ml) for an additional 3 d, respectively.

### Cell viability assay

The WT BMMs were seeded in a 96-well plate at a density of 1 × 10^3^ cells/well. After 24 h of culture, the cells were treated with various concentrations of the three *E. faecalis* LTAs for an additional 24 h. The effects of *E. faecalis* LTA on cell viability was evaluated using the Cell Counting Kit-8 (Sigma-Aldrich, St. Louis, MO, USA) according to the manufacturer’s instructions.

### TRAP staining

The WT BMMs and *Rbpj^ΔM/ΔM^* BMMs were seeded in 6-well plates at a density of 2 × 10^5^ cells/well, respectively. The cells were treated with the three *E. faecalis* LTAs and/or RANKL. TRAP staining was carried out using TRAP kit according to the manufacturer’s instruction (Sigma-Aldrich, St. Louis, MO, USA). The cells were washed twice with PBS and fixed with 4% paraformaldehyde for 30 min. Then the cells were rinsed thoroughly using pre-warmed deionized water. The staining mixing solution was prepared according to the manufacturer’s instructions. The cells were incubated in the staining solution, protected from light, at 37°C for 1 h. The cells were counterstained for 2 min in Hematoxylin solution and rinsed in tap water thoroughly. The TRAP-positive multinucleated cells were observed and counted under a light microscope.

### Gene expression analysis

Total RNA was extracted from WT BMMs after *E. faecalis* LTA treatment. The gene expression analyses were carried out using real-time PCR. Primer sequences were as follows: cathepsin K, 5′(CTGAAGATGCTTTCCCATATGTGGG)3′ and 5′(GCAGGCGTTGTTCTTATTCCGAGC)3′; TRAP, 5′(ACACAGTGATGCTGTGTGGCAACTC)3′ and 5′(CCAGAGGCTTCCACATATATGATGG)3′; MMP-9, 5′(GCCCTGGAACTCACACGACA)3′ and 5′(TTGGAAACTCACACGCCAGAAG)3′;c-Fos, 5′(ACGTGGAGCTGAAGGCAGAAC)3′ and 5′(AGCCACTGGGCCTAGATGATG)3′; Nfatc1, 5′(CAAGTCTCACCACAGGGCTCACTA)3′ and 5′(TCAGCCGTCCCAATGAACAG)3′; Notch1, 5′(GCTCCGAGGAGATCAACGAG)3′ and 5′(TTGACATCACCCTCACACCG)3′; Rbpj, 5′(CGGCCTCCACCCAAACGACT)3′ and 5′(TCCAACCACTGCCCATAAGATACA)3′; GAPDH 5′(ATGTGTCCGTCGTGGATCTGA)3′ and 5′(ATGCCTGCTTCACCACCTTCT)3′.

### ELISA assay

The supernatants were collected from WT BMMs after *E. faecalis* LTA treatment. The expression levels of TNF-α and IL-6 were analyzed with the corresponding ELISA kits (R&D systems, Minneapolis, MN, USA).

### Western blotting

The whole cell lysates were extracted from WT BMMs after *E. faecalis* LTA treatment. The phospho-p38, p38, phospho-ERK1/2 and ERK1/2 Abs (Cell Signaling Technology, Boston, MA, USA) were used to detect MAPK signaling pathways with Western blotting.

### Statistical analysis

Each experiment was conducted in triplicate and repeated at least three times. Data were presented as mean ± SD and analyzed by ANOVA. The threshold of statistical significance was set at P < 0.05.

## Results

### The effect of the three* E. faecalis *LTAs on the cell viability of BMMs

**Figure 1.**
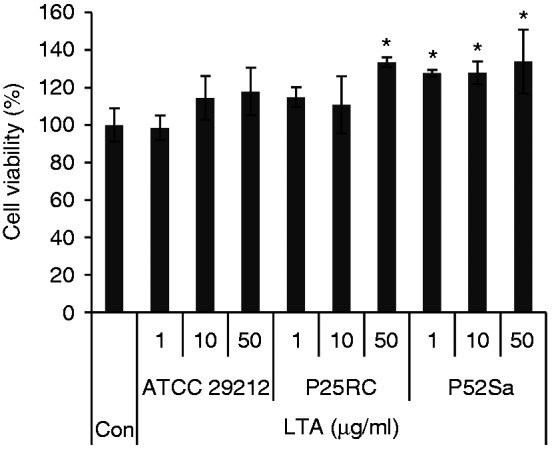
Effects of the three different *E. faecalis* LTAs on the cell viability of BMMs. WT BMMs were cultured with 40 ng/ml M-CSF and stimulated with the three different *E. faecalis* LTAs at various concentrations for 24 h. Data are presented as mean ± SD, *P < 0.05 compared with untreated cells. Con, untreated cells were set as control.

The WT BMMs were cultured with 40 ng/ml M-CSF and stimulated with the three different *E. faecalis* LTAs at various concentrations of 1 µg/ml, 10 µg/ml and 50 µg/ml for 24 h. The result showed that *E. faecalis* LTA could not inhibit the cell viability of osteoclast precursors. LTAs from *E. faecalis* P25RC (50 µg/ml) and P52Sa could increase the cell viability (Figure 1).

### *E. faecalis* LTAs inhibit RANKL-induced osteoclastogenesis

**Figure 2.**
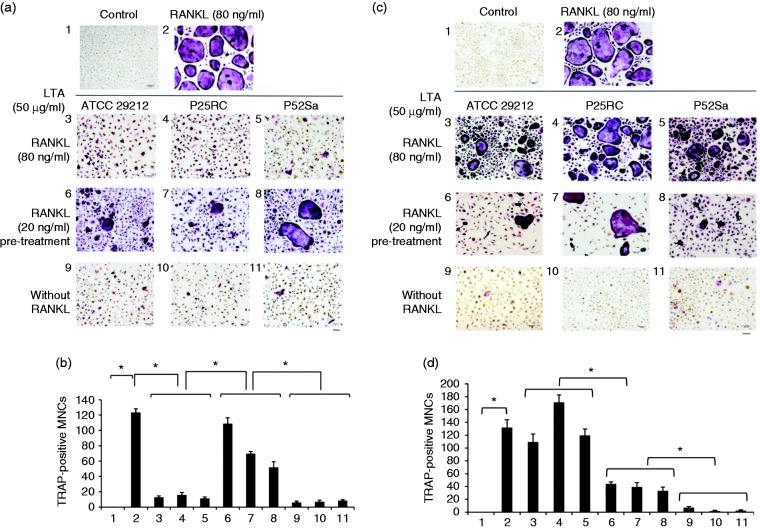
Osteoclast differentiation induced by the three *E. faecalis* LTAs in osteoclast precursors was evaluated with the TRAP staining assay. (a and b) WT BMMs and (c and d) *Rbpj^ΔM/ΔM^* BMMs were treated for 6 d in the presence of 40 ng/ml M-CSF with the three *E. faecalis* LTAs, RANKL, *E. faecalis* LTAs plus RANKL, or pre-treated with RANKL for 3 d prior to treatment with *E. faecalis* LTAs for an additional 3 d, respectively. The cells were then subjected to TRAP staining. TRAP staining assay was carried out and visualized at 100× magnification under an inverted bright field microscope. Bar, 100 µm. The numbers of TRAP-positive multinucleated cells with more than 3 nuclei were counted from 6 random fields of view at 40× magnification. 1, Untreated cells; 2, RANKL (80 ng/ml); 3, RANKL (80 ng/ml) and *E. faecalis* ATCC 29212 LTA (50 µg/ml); 4, RANKL (80 ng/ml) and *E. faecalis* P25RC LTA (50 µg/ml); 5, RANKL (80 ng/ml) and *E. faecalis* P52Sa LTA (50 µg/ml); 6, Pre-treated with RANKL (20 ng/ml) and *E. faecalis* ATCC 29212 LTA (50 µg/ml); 7, Pre-treated with RANKL (20 ng/ml) and *E. faecalis* P25RC LTA (50 µg/ml); 8, Pre-treated with RANKL (20 ng/ml) and *E. faecalis* P52Sa LTA (50 µg/ml); 9, *E. faecalis* ATCC 29212 LTA (50 µg/ml); 10, *E. faecalis* P25RC LTA (50 µg/ml); 11, *E. faecalis* P52Sa LTA (50 µg/ml). The mean and SD are shown. *P < 0.05 was considered statistically significant compared with the untreated cells or RANKL-only treated cells. MNCs, multinucleated cells.

The TRAP staining demonstrated that *E. faecalis* LTAs effectively inhibited osteoclast differentiation of WT BMMs in the presence of high-dose RANKL (80 ng/ml) and resulted in small, immature TRAP-positive osteoclasts with fewer nuclei, while *E. faecalis* LTAs could not inhibit osteoclast differentiation when WT BMMs were pre-treated with low-dose RANKL (20 ng/ml) (Figure 2(a) and 2(b)). However, *E. faecalis* LTAs induced osteoclast differentiation of *Rbpj^ΔM/ΔM^* BMMs and resulted in large osteoclasts with many nuclei in the presence of high-dose RANKL (80 ng/ml). In contrast, *E. faecalis* LTAs could induce WT BMMs and *Rbpj^ΔM/ΔM^* BMMs to form a few small, immature TRAP-positive osteoclasts independent of RANKL (Figure 2(c) and 2(d)).

### Gene expression of osteoclast differentiation induced by *E. faecalis* LTAs with and without RANKL

Gene expression levels of cathepsin K, TRAP and MMP-9 were significantly up-regulated to varying degrees when WT BMMs were treated with *E. faecalis* LTAs upon exposure to RANKL compared with the untreated control. Upon exposure to high-dose RANKL, expressions of the three osteoclast-related genes were markedly down-regulated by *E. faecalis* LTAs compared with RANKL treatment alone. However, compared with the untreated control, the expression level of cathepsin K was even all down-regulated by *E. faecalis* LTAs alone in WT BMMs.

**Figure 3.**
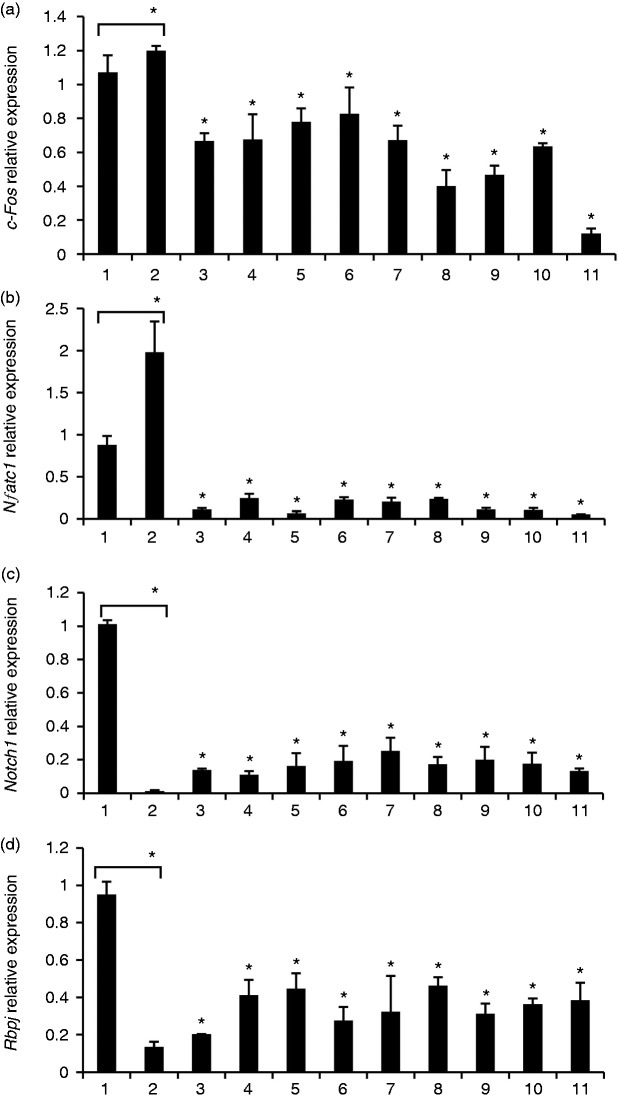
Gene expression analysis of osteoclast differentiation induced by *E. faecalis* LTA in osteoclast precursors. WT BMMs were treated for 6 d in the presence of 40 ng/ml M-CSF with the three *E. faecalis* LTAs, RANKL, *E. faecalis* LTAs plus RANKL, or pre-treated with RANKL for 3 d prior to treatment with *E. faecalis* LTAs for an additional 3 d, respectively. The gene expression levels of (a) c-Fos, (b) NFATc1, (c) Notch1 and (d) RBP-J were assayed using real-time PCR. The numbers of abscissa represent the same treatment groups as those in Figure 2. The mean and SD are shown. *P < 0.05 was considered statistically significant compared with untreated cells or RANKL-only treated cells.

Gene expression levels of c-Fos and NFATc1 were significantly up-regulated in WT BMMs only treated with high-dose RANKL compared with untreated control. In addition, gene expression levels of c-Fos and NFATc1 were significantly down-regulated in WT BMM treated with *E. faecalis* LTAs compared with cells only treated with high-dose RANKL (Figure 3(a) and 3(b)). On the contrary, gene expression levels of Notch1 and RBP-J were markedly down-regulated in WT BMMs treated only with high-dose RANKL compared with untreated control. Furthermore, gene expression levels of Notch1 and RBP-J were up-regulated in WT BMMs treated with *E. faecalis* LTAs compared with cells only treated with high-dose RANKL (Figure 3(c) and 3(d)).

### The pro-inflammatory effects of *E. faecalis* LTAs on osteoclast precursors

**Figure 4.**
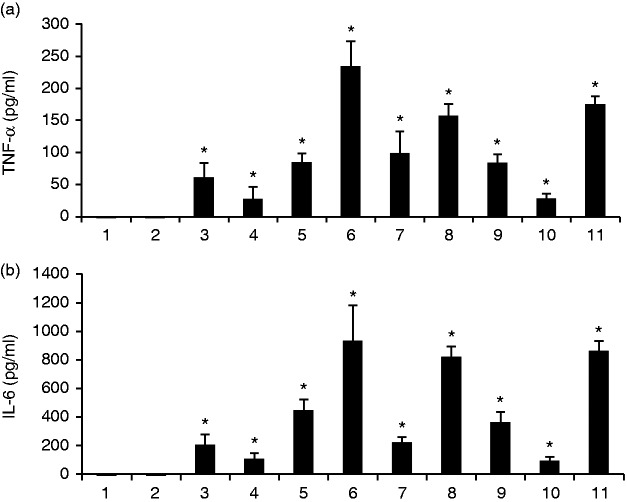
The pro-inflammatory effects of *E. faecalis* LTAs on osteoclast precursors. WT BMMs were treated for 6 d in the presence of 40 ng/ml M-CSF with the three *E. faecalis* LTAs, RANKL, *E. faecalis* LTAs plus RANKL, or pre-treated with RANKL for 3 d prior to treatment with *E. faecalis* LTAs for an additional 3 d, respectively. The secretory levels of (a) TNF-α and (b) IL-6 were assayed using ELISA. The numbers of abscissa represent the same treatment groups as those in Figure 2. The mean and SD are shown. *P < 0.05 was considered statistically significant compared with untreated cells.

The three *E. faecalis* LTAs significantly increased the levels of TNF-α and IL-6 in varying degrees compared with untreated control (Figure 4).

### Protein expression of osteoclast differentiation induced by* E. faecalis *LTAs with and without RANKL

**Figure 5.**
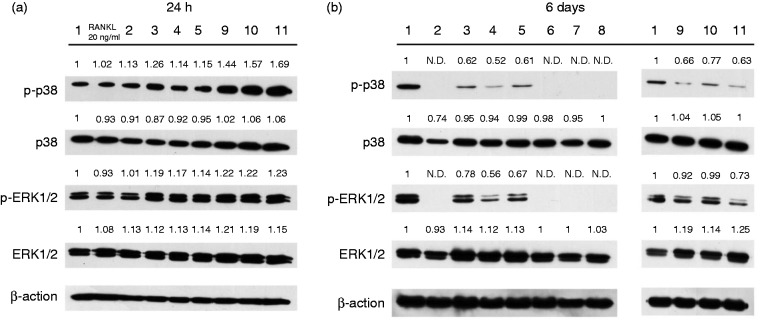
Protein expression analysis of osteoclast differentiation induced by *E. faecalis* LTA in osteoclast precursors. (a) WT BMMs were treated with the three *E. faecalis* LTAs, RANKL and *E. faecalis* LTAs plus RANKL for 24 h in the presence of 40 ng/ml M-CSF. (b) WT BMMs were treated for 6 d in the presence of 40 ng/ml M-CSF with the three *E. faecalis* LTAs, RANKL, *E. faecalis* LTAs plus RANKL, or pre-treated with RANKL for 3 d prior to treatment with *E. faecalis* LTAs for an additional 3 d, respectively. The numbers above the bold line represent the same treatment groups as those in Figure 2. The numerical values above the bands denote the relative density values.

Both p38 and ERK1/2 MAPK signaling pathways were activated after 24 h *E. faecalis* LTA treatment in WT BMM (Figure 5(a)). However, phosphorylation of both p38 and ERK1/2 MAPK could not be detected after 6 d with only high-dose RANKL treatment and RANKL pre-treatment prior to *E. faecalis* LTA treatment. Phosphorylation of both p38 and ERK1/2 MAPK were down-regulated by high-dose RANKL and *E. faecalis* LTA treatment and only *E. faecalis* LTA treatment (Figure 5(b)).

## Discussion

LTA is a key virulence factor in inflammatory process and is expressed exclusively on the surface of Gram-positive bacteria.^24^ The structure and function of LTA vary across different species.^25^ *E. faecalis* LTA is a typical D-alanyl-LTA with glycerophosphate backbone (Type 1).^5^^,^^26^ Structural microheterogeneity between various LTAs mainly lies in the D-alanylation rates, glycerolphosphate chain length, fatty acid composition and type of glycosyl substitution.^1^^,^^27^ These subtle differences may cause varying levels of inflammatory responses.^28^

In this study, *E. faecalis* LTA had no significant detrimental effect on cell viability, which was consistent with previous studies.^29^^,^^30^ On the contrary, LTAs from *E. faecalis* P25RC and P52Sa were observed to enhance cell viability after 24 h treatment, which may be explained in Figure 5(a). It was demonstrated that *E. faecalis* LTAs induced phosphorylation of p38 and ERK1/2 MAPKs after 24 h of treatment. MAPK signaling pathways are essential for cell proliferation and development.^31^ It is likely that the observed enhancement of cell viability might be caused by activation of MAPK signaling pathways.

Interestingly, this study showed that the three *E. faecalis* LTAs effectively inhibited osteoclast differentiation of WT BMMs in the presence of a high dose of RANKL and only resulted in the formation of some small immature TRAP-positive osteoclasts with fewer nuclei. The efficacy of RANKL on osteoclast differentiation was greatly reduced by *E. faecalis* LTAs. LTA, as a pro-inflammatory stimulus, could induce M1 polarization of macrophages. It has been demonstrated that M1 macrophages could attenuate osteoclastogenesis.^32^ A similar phenomenon was also observed in a previous report that *S. aureus* LTA inhibited osteoclastogenesis upon exposure to M-CSF and a low dose of RANKL (20 ng/ml).^30^ However, the inhibition of osteoclastogenesis by *E. faecalis* LTAs was removed in the *Rbpj^ΔM/ΔM^* BMM cell cultures, in which large amounts of osteoclasts with many nuclei were formed in the presence of a high dose of RANKL. It has been reported that RBP-J is responsible for M1 macrophage polarization.^17^ In this study, the RBP-J-deficiency inhibited M1 polarization of BMMs and revived osteoclastogenesis upon exposure to LTA and a high dose of RANKL. This thus suggests that *E. faecalis* LTA inhibits RANKL-induced osteoclast differentiation, at least partially via RBP-J. RBP-J plays an important role in the process of *E. faecalis* LTA inhibiting RANKL-induced osteoclastogenesis. In addition, the inhibitory effect of *E. faecalis* LTAs on osteoclastogenesis was decreased when WT BMMs were pre-treated with a low dose of RANKL (20 ng/ml), indicating that *E. faecalis* LTA mainly functions during the early stage of osteoclast differentiation. A few TRAP-positive immature small osteoclasts were formed by WT and *Rbpj^ΔM/ΔM^* BMMs with *E. faecalis* LTA treatment alone. *E. faecalis* LTA has a very weak effect in stimulating osteoclast differentiation, presumably through TNF-α, as reported previously.^33^

Gene expression levels of cathepsin K, TRAP and MMP-9 were suppressed by *E. faecalis* LTAs upon exposure to a high dose of RANKL, as compared with treatment with RANKL only. At the same time, *E. faecalis* LTAs had no significant effects in modulating the expression of the three osteoclast-related genes: cathepsin K, TRAP and MMP-9. Because LTAs were derived from different *E. faecalis* strains and had different structures, a few differences among groups with similar treatments could still be observed. *E. faecalis* LTAs inhibited the gene expression levels of c-Fos and NFATc1, while enhancing the gene expression levels of Notch1 and the negative regulator RBP-J, as compared with the RANKL treatment group. These results showed that *E. faecalis* LTAs could inhibit RANKL-induced osteoclast differentiation, which was consistent with the results of the TRAP staining analysis.

The ELISA analysis result showed that treatment with *E. faecalis* LTAs could significantly increase the production of pro-inflammatory cytokines, TNF-α and IL-6. TNF-α and IL-6 promote osteoclast differentiation.^34^ Therefore, the weak direct effects of *E. faecalis* LTAs on osteoclast differentiation might be associated with the secretion of TNF- α and IL-6.

*E. faecalis* LTAs significantly increased the phosphorylation of p38 and ERK1/2 after 24 h of treatment. MAPK signaling pathways are involved in the regulation of the production of inflammatory cytokines.^35^^,^^36^ In contrast, p38 and ERK1/2 MAPKs were not phosphorylated even after 6 d of treatment with RANKL only and RANKL pretreatment prior to exposure to *E. faecalis* LTAs. The activation of p38 and ERK1/2 MAPKs greatly decreased compared with the untreated WT BMMs. This might be related to the maturation and activation of osteoclasts. It has been previously reported that phosphorylation of p38 disappears during the differentiation of osteoclast precursors to mature osteoclasts.^37^ In this study, the phosphorylation of ERK1/2 was gradually reduced in osteoclast precursors during their differentiation to osteoclasts. Therefore, mature osteoclasts lost the capacity for phosphorylation of p38 and ERK1/2.

In conclusion, the present study shows that *E. faecalis* LTA may be a strong stimulator of inflammatory response, but a weak inducer of osteoclast differentiation, presumably due to the production of TNF-α and IL-6. *E. faecalis* LTA significantly inhibited RANKL-induced osteoclastogenesis via RBP-J.

## Declaration of conflicting interests

The author(s) declared no potential conflicts of interest with respect to the research, authorship, and/or publication of this article.
